# The social cost of chronic kidney disease in Italy

**DOI:** 10.1007/s10198-016-0830-1

**Published:** 2016-10-03

**Authors:** Giuseppe Turchetti, S. Bellelli, M. Amato, S. Bianchi, P. Conti, A. Cupisti, V. Panichi, A. Rosati, F. Pizzarelli, On Behalf of the Tuscany CKD Study Group, A. Casani, G. Rosso, A. Capitanini, C. Del Corso, S. Aterini, L. Bozzoli, G. Grazi, D. Guzzo, P. Paparatto, A. Baronti, G. Pratesi, G. Garosi, E. Sansoni, K. Natflos, A. Sidoti, M. L. Lusini, E. Duranti, C. Mura, C. Gabrielli, P. Dattolo, A. Scatena

**Affiliations:** 1 Institute of Management, Scuola Superiore Sant’Anna, piazza Martiri della Libertà 33, 56127 Pisa, Italy; 2 Nephrology Unit, Azienda USL Toscana centro (ex-AUSL 4), piazza Ospedale 5, 59100 Prato, Italy; 3 Nephrology Unit, Azienda USL Toscana nord ovest (ex-AUSL 6), viale Alfieri 36, 57124 Livorno, Italy; 4 Nephrology Unit, Azienda USL Toscana sud est (ex-AUSL 9), via Cimabue 109, 58100 Grosseto, Italy; 5 Department of Clinical and Experimental Medicine, University of Pisa, Lungarno Pacinotti 43, 56126 Pisa, Italy; 6 Nephrology Unit, Azienda USL Toscana nord ovest (ex-AUSL 12), via Aurelia 335, Lido di Camaiore, Lucca, Italy; 7 Nephrology Unit, Azienda USL Toscana nord ovest (ex-AUSL 2), via per S.Alessio, Monte San Quirico, Lucca, Italy; 8 Nephrology Unit, Azienda USL Toscana centro (ex-AUSL 10, Santa Maria Annunziata Hospital), piazza Santa Maria Nuova 1, 50122 Firenze, Italy

**Keywords:** Cost of illness, Chronic kidney disease, CKD stages 4 and 5, Direct costs, Indirect costs, Italy, I15 (health and economic development)

## Abstract

This study aims to estimate the mean annual social cost per patient with chronic kidney disease (CKD) by stages 4 and 5 pre-dialyses and cost components in Italy. The multicenter cross-sectional study included all adult outpatients in charge of the 14 main Nephrology Centers of Tuscany Region during 7 weeks from 2012 to 2013. Direct medical costs have been estimated using tariffs for laboratory tests, diagnostic exams, visits, hospitalization and prices for drugs. Non-medical costs included expenses of low-protein special foods, travel, and formal and informal care. Patients’ and caregivers’ losses of productivity have been estimated as indirect costs using the human capital approach. Costs have been expressed in Euros (2016). Totals of 279 patients in stage 4 and 205 patients in stage 5 have been enrolled. The estimated mean annual social cost of a patient with CKD were €7422 (±€6255) for stage 4 and €8971 (±€6503) for stage 5 (*p* < 0.05). Direct medical costs were higher in stage 5 as compared to stage 4; direct non-medical costs and indirect costs accounted, respectively, for 41 and 5 % of the total social cost of CKD stage 4 and for 33 and 9 % of CKD stage 5. In Italy, the overall annual social cost of CKD was €1,809,552,398 representing 0.11 % of the Gross Domestic Product. Direct non-medical costs and indirect costs were weighted on the social cost of CKD almost as much as the direct medical cost. Patients, their families and the productivity system sustain the burden of the disease almost as much as the healthcare system.

**Electronic supplementary material:**

The online version of this article (doi:10.1007/s10198-016-0830-1) contains supplementary material, which is available to authorized users.

## Introduction

Chronic kidney disease (CKD) represents a major public health concern with a great economic burden [1].

In 2002, the National Kidney Foundation’s Kidney Disease Outcomes Quality Initiative (NKF KDOQI) introduced a conceptual model for the definition and classification of CKD [2]. CKD was classified into five stages based on the level of glomerular filtration rate (GFR), with higher stages (3–5) representing lower GFR levels and increasing severity of renal damage, eventually necessitating dialysis. CKD stage 4 occurs when the residual renal function was reduced from 30 to 15 %, whereas in CKD stage 5 the residual renal function dropping below 15 % identifies the end-stage of the disease. In CKD stage 4, several signs, symptoms or complications of renal insufficiency begin to arise, namely anemia, loss of appetite, uncontrolled hypertension, metabolic acidosis, secondary hyperparathyroidism, hyperphosphatemia or iperkalemia. These clinical manifestations were much more prevalent in CKD stage 5 and represented the indication to start dialysis. This framework has had enormous effects on clinical practice, research and public health policy.

Across European study populations, the adjusted CKD stages 3–5 prevalence varied between 1.0 % [95 % confidence interval (95 % CI) 0.7–1.3 %] in central Italy and 5.9 % (95 % CI 5.2–6.6 %) in northeast Germany [3].

In Italy, CKD in stage 4 and stage 5 affects, respectively, 0.3 and 0.15 % of the general population [4]. The risk of having CKD increases after 50 years of age and is most common among adults older than 70 years [5, 6].

The disease is characterized by an interaction of risk factors and comorbidities, particularly diabetes, arterial hypertension, cardiovascular disease and a family history of CKD. Approximately 1 in 3 adults with diabetes and 1 in 5 adults with high blood pressure have CKD [5]. Diabetes occurs in 27 % of CKD end-stage 3 cases and in 40 % of patients with CKD stages 4 and 5 [7]; 89 % of patients with CKD stages 3–5 have hypertension [8].

The progressive reduction of renal function leads to a decline of patient quality of life. The health-related quality of life (HRQoL) measures were reduced in CKD patients in proportion to the severity grade of the disease. A significant decline in cognitive function and sleep disturbances, pain, and physical and mental dysfunctions have been observed in patients with CKD by stage of disease [9]. In addition, the debilitating nature of the disease may affect the working ability of patients leading to absenteeism and loss of employment [10].

In an healthcare setting characterized by a limited number of resources, it is important to identify, measure and estimate disease-associated costs as an operational tool aimed to support the policymakers in deciding health policy strategies and resources allocation [11, 12]. Due to the epidemiological burden of the disease and the progressive deterioration of kidney function leading to dialysis and renal transplant, these considerations are becoming more and more relevant.

Cost of illness (COI) studies answer to the need of quantifying the economic burden of CKD on the society by evaluating the major cost components of the disease, such as direct medical and non-medical costs and indirect costs. Direct costs evaluate the use of resources directly related to CKD, while indirect costs are related to loss of productivity of the patient and his/her caregivers caused by the disease [13].

The economic burden of the CKD in pre-dialyses stages has not been widely investigated in the literature. Among the European countries, the mean annual direct medical cost per patient was €3581 in Germany for moderate and severe CKD [14]. Hospitalizations represent the main cost component of the total direct medical costs, followed by drugs and physician visits. In Italy, the direct medical annual cost per patient was estimated at €890 in stage 3, €3392 in stage 4 [4] and €13,752 in stage 5 [15]. Moreover, costs of CKD increase in the presence of comorbidities, such as anemia [16], diabetes and cardiovascular diseases [17] and secondary hyperparathyroidism [18].

The substantial social economic burden of CKD in pre-dialyses stages has not been widely investigated in the scientific literature. A limited number of studies aimed at quantifying the social cost of a patient with CKD in pre-dialyses stages are available with a high level of heterogeneity and incompleteness in cost estimates. In most studies, the direct medical component of the costs has been estimated, while missing or insufficient estimates have been reported for direct non-medical costs and indirect costs.

The aim of this study is to estimate the mean annual social cost per patient with CKD by stage (4 and 5 pre-dialyses) and by cost component (direct medical and non-medical costs and indirect costs) in Italy.

## Methods

A cross-sectional cost of illness study was conducted by consecutively enrolling all adult outpatients (age ≥18 years) who agreed to participate to the study. Patients with pre-dialyses CKD stage 4 (estimated glomerular filtration rate, eGFR, 15–29 ml/min/1.73 m^2^) and stage 5 (eGFR <15 ml/min/1.73 m^2^ not yet on dialysis) in the charge of 14 Hospital Centers in Tuscany Region (Italy) were enrolled during 7 weeks. The study was carried out between March 2012 and March 2013.

**Fig. 1:**
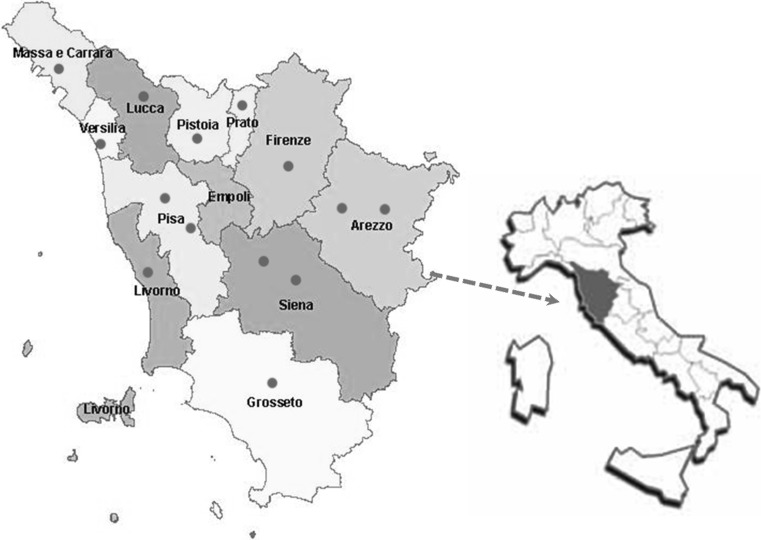
Centers enrolled in the study, Tuscany Region, Italy

Centers participating in the study are the main Nephrology Centers of Tuscany uniformly distributed throughout the Region (Fig. 1). According to normal clinical practice, patients with CKD stages 4 or 5 pre-dialyses were followed up in the outpatient clinic dedicated to nephropathic patients. According to the international classification of CKD provided by the National Kidney Foundation’s clinical practice guidelines, patients were defined in CKD stages 4 or 5 pre-dialyses basing on the level of GFR estimated by the Modification of Diet in Renal Disease (MDRD) formula [2].

A Case Report Form (CRF) aimed at collecting patient data was drawn by the Health Economics Group of the Institute of Management, Scuola Superiore Sant’Anna (Pisa, Italy) in collaboration with nephrologists involved in the study. The CRF included a clinical section and a socio-demographic and economic section, and it was submitted, respectively, to the clinician and to the patient at the time of the outpatient specialist visit.

The clinical section aimed to collect clinical data of the patient and to identify and evaluate the direct medical costs components. The patient’s stage of CKD, assessed by the MDRD formula, and the presence and type of comorbidities were collected. The medical components of direct costs have been retrospectively evaluated by asking the clinician to provide, from hospital records, the patient’s number and type of specialist visits, laboratory tests, diagnostic exams, drug consumption and hospitalizations. Drugs assumed by patients for chronic treatments were recorded with their commercial name, package, active principle and prescribed dosage. Hospitalizations included inpatient care and day-hospital procedures by collecting the ICD9-CM principal diagnosis at discharge, as well as the corresponding Diagnosis Related Group (DRG), number of days of hospital stay and number of day-hospital admissions. The economic information collected by the hospital patient records of each center involved in the study were related to the 12 months before filling in the CRF.

The socio-demographic section recorded patient information about gender, age, civil status and education attainment, as well as data aimed at estimating the non-medical component of the direct costs and indirect costs. The non-medical component of direct costs have been evaluated by asking to patient: (1) out-of-pocket expenses for transport (e.g., private and public transport used for going to the hospital), (2) costs for diet (e.g., low-protein special foods) including out-of-pocket expenses and reimbursement provided by the National Health System (NHS), and (3) number of weekly hours of help in domestic work provided by housekeepers or caregivers for estimating, respectively, paid domestic help or informal care costs. Information for estimating indirect costs such as the patient’s and his/her main caregiver’s loss of productivity have been estimated by questions on working conditions. The type of working sector, number of working days lost and working hours lost due to the disease in the prior 3 months were asked of working patients.

Missing data have been identified by specific queries that have been submitted to each Center in order to fulfill the missing information.

### Economic evaluation of the social cost of CKD

A societal perspective was taken to identify the economic burden of CKD by stage of disease.

The economic evaluation has been performed for direct medical cost component using the corresponding Tuscany Region’s outpatient tariffs for laboratory tests, diagnostic exams, first visit and follow-up visits [19]. The cost of each pharmacological treatment assumed by the patient was calculated by multiplying daily dosage by cost unit and by the number of treatment days in the year of reference. The cost units of each drug has been calculated by merging the information collected on the drug’s commercial name and package with the corresponding NHS sale price and retail price [20, 21]. Hospitalization tariffs, such as the DRG-specific inpatient tariffs and day-hospital tariffs, have been collected by the Accounting Department of each Center involved in the study.

The direct non-medical component costs concerning the costs for transport and diet have been collected by CRF. Out-of-pocket expenses of paid public or private transport were asked of the patient referring to the cost for a round trip from home to the Hospital in order to perform the specialist visit. The overall annual transport cost was then calculated by assuming that the patient spent the same amount for going to the Hospital for performing all specialist visits, for inpatient care and day-hospital admissions in the year of reference. Caregiver’s transport expenses were assumed equal to those incurred by the patient for public transport. Costs for low-protein special foods have been collected by asking the patient the mean monthly out-of-pocket expenses and the mean monthly reimbursement provided by the NHS. The paid domestic help has been evaluated by investigating the number of weekly hours needed for domestic help due to his/her physical condition. The yearly working hours requested for domestic help were then calculated and multiplied by the hourly wage of a housekeeper (distinguishing whether or not living with the patient) based on the National Agreement for Home Labour Service [22]. Time lost for informal care, such as unpaid domestic help provided by caregivers, has been evaluated based on the wage that would have been paid to an housekeeper [23].

Patient and caregiver indirect costs were determined through the human capital approach. The productivity losses for working patients were calculated by multiplying the working hours lost due to CKD over the past year by the average hourly earned income in different working sectors. The working hours lost due to CKD in the previous year were computed from the CRF data as the number of full working days lost and the number of working hours lost in the last 3 months by the patients. It has been assumed that 8 working hours were lost by full-time workers in the private sector or by the self-employed and 7.2 working hours were lost by full-time employers in the public sector. The mean hourly gross earnings of a full-time employer were collected by type of working sector (private or public) according to data from the National Institute of Statistics (ISTAT) [24, 25]. The loss of productivity of working caregivers has been evaluated by multiplying the working hours lost by the mean hourly gross earnings of a full-time employer [24, 25]. The working time lost by the caregiver has been assumed to be equal to the yearly mean number of working hours lost by patient due to CKD. Leisure time lost with no direct effects on economic productivity has been assumed for non-working patients and caregivers such as housewives, out-of-work patients, retirees and students according to the economic literature [23].

A sensitivity analysis has been conducted for estimating indirect costs by appling the replacement cost approach to non-working patients and caregivers. The replacement cost approach was applied to housewife patients by evaluating their productivity losses with the current market value of a housekeeper, i.e. the hourly wage of a housekeeper based on the National Agreement for Home Labour Service [22]. The housewives’ time lost has been assumed to be equal to the mean yearly hours lost by CKD patients. Time lost by patients who retired due to CKD and belong to the working age class (i.e. <65 years old) have been estimated by considering the net yearly earnings lost after social security benefits due to the disease [26]. The replacement cost approach was applied to non-working caregivers, such as housewives, retirees, students and out-of-work patients. Their productivity losses have been computed by multiplying the yearly mean number of working hours lost by patients due to CKD by the hourly wage of a housekeeper [22].

### Statistical analyses

Statistical analyses have been performed by stratifying the sample of CKD patients by stage of disease, i.e. CKD stage 4 and CKD stage 5 pre-dialyses.

Descriptive statistics have been provided for summarizing the socio-demographic, clinical and economic characteristics of patients as a proportion for categorical variables and mean (±SD, standard deviation) or median (IQR, inter-quartile range), respectively, for normally or non-normally distributed continuous data. Distributions of continuous variables have been tested with the Shapiro–Wilk normality tests. Differences in distribution of patients variables were assessed using the Fisher’s exact test or the Pearson’s Chi-squared test for categorical variables and the two-sided two-sample *t* test or two-sample Wilcoxon rank-sum (Mann–Whitney) test, respectively, for normal and non-parametric continuous variables. Two-sided *p* values <0.05 were deemed statistically significant.

The distribution of costs has been found, as expected, to be positive and right-skewed with few patients requiring much more healthcare resources than the average, incurring very high costs and creating a long right tail. Costs have been expressed as mean (±SD) with the purpose of being helpful to the healthcare policy makers who require information on the overall social costs of the disease, which can be obtained by multiplying the mean cost by the total number of sick patients [27]. Mean annual unadjusted cost data per patient have been calculated by type of cost component in order to estimate the burden of each cost item on the overall social cost. Multivariate analyses have been performed for estimating the association of patients’ socio-demographic and clinical characteristics with the CKD social cost and their incremental or marginal effects. The generalized linear model (GLM) framework offers considerable flexibility in modeling cost data by allowing the mean and variance function of expenditures to be directly specified. GLM has been performed with its most commonly used specification as the log link with a gamma variance, confirmed by the modified Park test [28].

Costs were expressed in Euros (2016).

Data analyses were performed using the STATA v.12 software (Stata, College Station, TX, USA).

## Results

A total of 484 patients were enrolled in the study, of whom 279 had CKD in stage 4 (median e-GFR: 20, IQR: 17–23) and 205 were in stage 5 pre-dialyses (median e-GFR: 11, IQR: 9–13). Totals of 268 (96.1 %) patients with CKD in stage 4 and 193 (94.2 %) in stage 5 had comorbidities (*p* = 0.329).

Twenty-two (7.9 %) patients with CKD in stage 4 and 29 (14.2 %) patients in stage 5 were workers in the private sector or self-employed (19, 86.4 %, and 25, 86.2 %, respectively in CKD stages 4 and 5) or in public sector (3, 13.6 %, and 4, 13.8 %). No statistically significant differences were found between the median number of hours of work lost by working patients in stages 4 and 5.

By comparing the working status of CKD stage 4–5 patients with that of the age-matched general population of Tuscany [29], it has been observed that the employment rate of CKD study’s working age population was significantly lower (39.0 vs. 63.7 %). The high prevalence of retiree (45.0 %) in the working age study sample due to the presence of patients receiving early retirement pensions and disability pensions could explain the low proportion of workers among the study patients.

Twenty-two (7.9 %) and 14 (6.8 %) patients with CKD in stages 4 and 5 used health transports such as ambulances or social welfare transports for going to the Center without paying money, while private transports were used for 242 (94.9 %) and 182 (96.3 %) patients and public transports for 13 (5.1 %) and 6 (3.2 %) patients in stages 4 and 5 (*p* = 0.328), respectively.

More than half the patients in stages 4 and 5 needed the presence of caregivers for going to the specialist visit. Among patients with caregivers, 164 (88.7 %) and 118 (84.3 %) patients went with one caregiver, while 21 (11.3 %) and 22 (15.7 %) went with two or more caregivers (*p* = 0.280), respectively. Caregivers were women in 125 (67.6 %) and 103 (73.6 %) cases, respectively, in stage 4 and stage 5 (*p* = 0.270) and they had a median age of 58.9 years (IQR: 48.8–68.8) and 60.8 (IQR: 49.8–71.0), *p* = 0.393. In most cases, caregivers were workers (77, 41.6 % and 54, 38.6 %) followed by retirees (62, 33.5 % and 49, 35.0 %) and housewives (32, 17.3 % and 27, 19.3 %), respectively, for patients in stages 4 and 5.

More than 1 in 3 patients needed domestic help due to CKD. Domestic help was provided by caregivers in 71 of 105 (67.6 %) and 59 of 79 (74.7 %) cases for stages 4 and 5 (*p* = 0.276), with a mean number of hours per week equal to 5.8 (SD, ±11.0) and 5.2 (SD, ±10.1), *p* = 0.545, respectively.

Most patients consumed low-protein special foods for CKD with a statistically significant prevalence in stage 5. Among patients consuming these special foods, the Italian NHS provided reimbursement for 155 (78.3 %) and 146 (83.0 %) cases in stages 4 and 5 (*p* = 0.255).

Data were shown in Table 1.

**Table 1:** Socio-demographic, clinical and economic characteristics of CKD patients by stages 4 and 5 pre-dialyses

	CKD stage 4		CKD stage 5		Overall		*p*
**Gender (** ***n*** **, %)**							
Male	177	63.4 %	126	61.5 %	303	62.6 %	0.657^c^
Female	102	36.6 %	79	38.5 %	181	37.4 %	
**Age (years) at enrollment**							
Median, IQR	75.5	67.7–82.2	73.1	65.7–80.4	74.5	66.7–81.5	0.063^d^
**e-GFR (MDRD)**							
Median, IQR	20	17–23	11	9–13	16	12–21	0.000^d^
**Comorbidity (** ***n*** **, %)**							
No	11	3.9 %	12	5.6 %	23	4.8 %	0.329^c^
Yes	268	96.1 %	193	94.2 %	461	95.3 %	
**Cardiovascular disease (** ***n*** **, %)**							
No	145	52.0 %	125	61.0 %	270	55.8 %	0.049^c^
Yes	134	48.0 %	80	39.0 %	214	44.2 %	
**Diabetes mellitus (** ***n*** **, %)**							
No	181	64.9 %	132	64.4 %	313	64.7 %	0.912^c^
Yes	98	35.1 %	73	35.6 %	171	35.3 %	
**Civil status (** ***n*** **, %)**							
Single	20	7.2 %	9	4.4 %	29	6.0 %	0.155^c^
Cohabitation/married	179	64.2 %	148	72.2 %	327	67.6 %	
Separated/divorced/widower	79	28.3 %	48	23.4 %	127	26.2 %	
**Education (** ***n*** **, %)**							
No qualification	16	5.7 %	5	2.4 %	21	4.3 %	0.028^c^
Primary degree	148	53.1 %	91	44.4 %	239	49.4 %	
Secondary degree^a^	101	36.2 %	100	48.8 %	201	41.5 %	
University degree^b^	11	3.9 %	7	3.4 %	18	3.7 %	
**Working condition (** ***n*** **, %)**							
Worker	22	7.9 %	29	14.2 %	51	10.5 %	0.064^c^
Out of work (unemployed)	2	0.7 %	4	2.0 %	6	1.2 %	
Student	1	0.4 %	0	0.0 %	1	0.2 %	
Housewife	16	5.7 %	15	7.3 %	31	6.4 %	
Retiree	238	85.3 %	157	76.6 %	395	81.6 %	
**Number of working hours lost per worker patient/year**							
Median, IQR	84	16–320	86	12–320	86	16–320	0.977^d^
**Kind of transport used by patient to get to the Center (** ***n*** **, %)**							
Health transport (ambulance/social welfare services)	22	7.9 %	14	6.8 %	36	7.4 %	0.667^c^
Non health (private/public) transport	255	91.4 %	189	92.2 %	444	91.7 %	
**Need of caregiver (** ***n*** **, %)**							
No	93	33.3 %	64	31.2 %	157	32.4 %	0.630^c^
Yes	185	66.3 %	140	68.3 %	325	67.2 %	
**Number of hours lost by caregiver per patient/year**							
Mean, ±SD	160.4	±113.9	165.4	±112.1	162.5	±113.1	0.630^e^
**Need of domestic help due to the disease (** ***n*** **, %)**							
No	173	62.0 %	126	61.5 %	299	61.8 %	0.864^c^
Yes	105	37.6 %	79	38.5 %	184	38.0 %	
**Number of hours required for domestic help per week**							
Mean, ±SD	5.8	±11.0	5.2	±10.1	5.5	±10.6	0.545^e^
**Patients consuming low-protein special food (** ***n*** **, %)**							
No	77	27.6 %	26	12.7 %	103	21.3 %	0.000^c^
Yes	198	71.0 %	176	85.9 %	374	77.3 %	
**Overall (** *n* **, %)**	279	100.0 %	205	100.0 %	484	100.0 %	

Discrepancies in totals are due to missing values

*CKD* chronic kidney disease,* GFR* glomerular filtration rate,* MDRD* Modification of Diet in Renal Disease,* IQR* inter-quartile range,* SD* standard deviation

^a^Primary degree refers to the “Licenza elementare” in Italy

^b^Secondary degree refers to the lower secondary degree “Diploma di scuola media inferiore” and to upper secondary degree “Diploma di scuola media superiore” (i.e. high school diploma) in Italy

^c^Pearson *X* ^2^ test

^d^Two-sample Wilcoxon rank-sum (Mann–Whitney) test

^e^Two-sample *t* test

Statistically significant differences have been found in healthcare resources consumption between the CKD stage 4 and CKD stage 5 patients groups. For CKD stage 4 and stage 5, respectively, the yearly mean number of tests performed per patient was 68.6 ± 51.5 (SD) and 93.5 ± 54.6, *p* = 0.000 (overall, it was 79.1 ± 54.2); the yearly mean number of specialist visits performed per patient was 6.0 ± 4.4 and 7.6 ± 4.1, *p* = 0.000 (overall, it was 6.7 ± 4.3); the yearly mean number of diagnostic exams performed per patient was 3.4 ± 3.2 and 4.2 ± 3.4, *p* = 0.015 (overall, it was 3.7 ± 3.3). Sixty-nine (24.7 %) patients in stage 4 and 69 (33.7 %) patients in stage 5 had almost one hospitalization in a year (*p* = 0.032) and 10 (3.6 %) and 12 (5.9 %) had almost one day-hospital access in a year (*p* = 0.236). The unadjusted estimated mean annual cost for hospitalizations was €1,298.9 ± €3233.3 per patient with CKD in stage 4 and €1664.5 ± €2806.1 in stage 5, *p* = 0.185. Day hospital access amounted to €63.5 ± €454.4 per patient with CKD in stage 4 and €231.1 ± €1064.4 in stage 5, *p* = 0.035. The mean number of hospitalization days or day-hospital access per patient/year was 2.9 ± 6.7 and 4.4 ± 8.1, respectively, for CKD stages 4 and 5, *p* = 0.025 (overall, it was 3.5 ± 7.3. Concerning drugs, a statistically significant difference was observed between the mean number of drugs by patients with CKD in stage 4 (7.6 ± 3.0) and stage 5 (8.5 ± 3.2), *p* = 0.002 (overall, it was 8.0 ± 3.1).

The unadjusted estimated mean annual social costs of a patient with CKD were €7421.6 ± €6255.2 for stage 4 and €8971.0 ± €6503.3 for stage 5 with a statistically significant difference (*p* = 0.008). Costs increase with the worsening of the disease. Direct non-medical costs and indirect costs accounted, respectively, for 41 and 5 % of the total social cost for CKD in stage 4 and for 33 and 9 % in stage 5.

By applying the replacement cost approach for estimating indirect costs for housewives and retiree patients and for non-working caregivers, a consequent significant increase of cost components has been observed. Results of the sensitivity analysis showed that the overall loss of productivity of patients and caregivers amounted to €2466.5 ± €2765.2 for CKD stage 4 and €2978.3 ± €3950.5 for CKD stage 5 (overall €2683.3 ± €3325.2). Overall, the unadjusted estimated mean annual social costs of a patient with CKD were €9514.8 ± €6545.9 for stage 4 and €11,152.4 ± €7644.0 for stage 5 with a statistically significant difference (*p* = 0.012). Direct non-medical costs and indirect costs accounted, respectively, for 32 and 26 % of the total social cost for CKD in stage 4 and for 26 and 27 % in stage 5 (Table 2).

**Table 2:** Unadjusted mean social annual cost data per patient by cost component and by CKD stages 4 and 5 pre-dialyses (Euro 2016)

Cost component	CKD stage 4			CKD stage 5			Overall			*p* ^a^
	Mean	SD	%	Mean	SD	%	Mean	SD	%	
**Direct medical cost**										
Laboratory test	281.5	±222.6	3.8	392.6	±233.2	4.4	328.5	±233.4	4.1	
Specialist visit	101.6	±69.9	1.4	126.3	±66.6	1.4	112.1	±69.5	1.4	
Diagnostic exam	157.4	±167.4	2.1	186.0	±176.0	2.1	169.5	±171.5	2.1	
Hospitalization	1362.4	±3250.6	18.4	1895.6	±3100.4	21.1	1588.2	±3195.5	19.7	
Drugs	2075.1	±2016.3	28.0	2629.3	±2173.7	29.3	2309.8	±2100.2	28.6	
Total	3978.1	±4187.0	53.6	5229.8	±4161.2	58.3	4508.2	±4217.5	55.8	0.001
**Direct non-medical cost**										
Out-of-pocket cost of diet	154.7	±421.9	2.1	170.4	±420.7	1.9	161.3	±421.1	2.0	
Reimbursement of diet	447.8	±285.4	6.0	539.0	±207.1	6.0	486.4	±258.9	6.0	
Transport of patient and caregiver	60.4	±80.4	0.8	75.5	±84.5	0.8	66.8	±82.4	0.8	
Paid domestic help	701.7	±2378.0	9.5	508.3	±2208.6	5.7	619.8	±2307.4	7.7	
Informal care	1705.6	±3516.0	23.0	1651.1	±3458.1	18.4	1682.5	±3488.1	20.8	
Total	3070.2	±3984.9	41.4	2944.3	±3958.0	32.8	3016.9	±3969.9	37.3	0.731
**Indirect costs**										
Loss of productivity of patient and caregivers	373.4	±2142.6	5.0	796.7	±3297.0	8.9	552.7	±2697.6	6.8	0.109
**Overall**	7421.6	±6255.2	100.0	8971.0	±6503.3	100.0	8077.8	±6400.9	100.0	0.008

*CKD* chronic kidney disease, *SD* standard deviation

^a^Two-sample *t* test

**Fig. 2:**
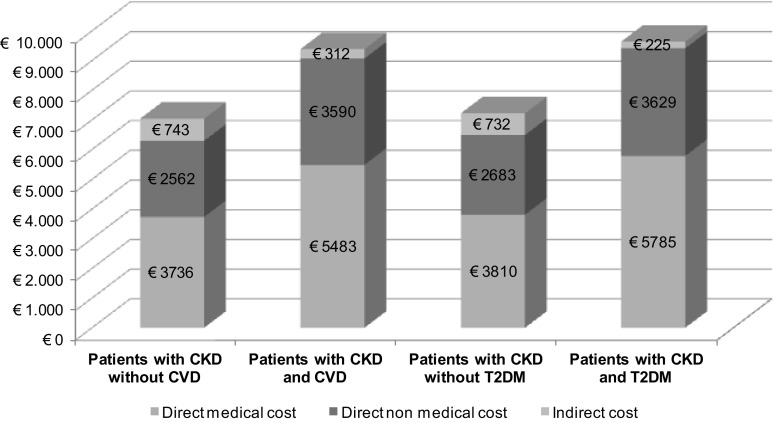
Unadjusted mean social annual cost per patient by CKD-related comorbidities and cost components, as direct medical, non-medical and indirect costs, Euro 2016

By analyzing CKD-related comorbidites, it was found that CVD and T2DM significantly increased the social cost of CKD: the raw estimated mean annual social costs were €9385 ± €7023) per patient with CKD and CVD versus €7041 ± €5663 per patient with CKD without CVD (*p* = 0.000) and €9639 ± €7108) per patient with CKD and T2DM versus €7225 ± €5817 per patient with CKD without T2DM (*p* = 0.000). Direct non-medical costs and indirect costs accounted for 42 % of the overall social cost for CKD and CVD and 40 % for CKD and T2DM (Fig. 2).

Effects of patients socio-demographic and clinical characteristics on social costs components, as direct medical, non-medical and indirect costs, have been investigated by multivariate generalized linear models performed by CKD stage group (stages 4 and 5 pre-dialyses and overall), adjusting for age, gender, diabetes mellitus, and cardiovascular disease (Table 3). Multivariate analyses showed a statistically significant effect of the presence of comorbidities such as diabetes mellitus and cardiovascular disease on direct medical costs and on the overall social cost. The estimated incremental effects of having diabetes mellitus and cardiovascular disease on direct medical costs were respectively €1976.1 (95 % CI €939.7–€3012.5, *p* = 0.000) and €1392.6 (95 % CI €531.9–€2253.2, *p* = 0.002) for CKD stage 4 and €1058.4 (95 % CI €129.8–€2246.7, *p* = 0.081) and €2083.3 (95 % CI €898.7–€3267.9, *p* = 0.001) for CKD stage 5. Patients with age lower than 74 years had unadjusted mean direct medical cost greater than those with age >74 years (€4877.2 ± €4598.4 vs. €4162.8 ± €3803.7, *p* = 0.062), due to statistically significant higher costs of laboratory tests, diagnostic exams and day hospital cares. Consistently, in the multivariate analyses, overall in stages 4 and 5, the adjusted annual direct medical cost of patients with age greater than 74 years was lower than that of patients with age ≤74 years (€960.9, 95 % CI: €1652.8–€268.9). Focusing on direct non-medical and indirect costs in CKD stage 5, gender has been found to be positively associated: the annual direct non-medical and indirect costs for women were €1810.5 (95 % CI €309.9–€3311.1, *p* = 0.018) greater than men.

**Table 3:** Incremental effects estimated by multivariate GLM regression (log link, gamma family) adjusted for age, gender, diabetes mellitus and cardiovascular disease by CKD stages 4 and 5 pre-dialyses and by cost components, as direct medical, non-medical and indirect costs, Euro 2016

Patient’s characteristics	CKD stage 4			CKD stage 5			Overall		
	Incremental effects	95 % CI	*p*	Incremental effects	95 % CI	*p*	Incremental effects	95 % CI	*p*
**Direct medical costs**									
Age (>74 years)	−941.4	−1820.5 to 62.2	0.036	−775.4	−1840.5 to 289.7	0.154	−960.9	−1652.8 to 268.9	0.006
Female gender	−696.8	−1537.7 to 144.0	0.104	−87.4	−1198.0 to 1023.3	0.877	−409.8	−1114.6 to 295.0	0.254
Diabetes mellitus	1976.1	939.7 to 3012.5	0.000	1058.4	−129.8 to 2246.7	0.081	1602.9	823.3 to 2382.5	0.000
Cardiovascular disease	1392.6	531.9 to 2253.2	0.002	2083.3	898.7 to 3267.9	0.001	1568.9	843.6 to 2294.1	0.000
**Direct non-medical and indirect costs**									
Age (>74 years)	960.8	−71.2 to 1992.9	0.068	7.7	−1319.0 to 1334.4	0.991	489.6	−347.1 to 1326.3	0.251
Female gender	622.5	−412.0 to 1657.0	0.238	1810.5	309.9 to 3311.1	0.018	1187.8	315.7 to 2059.8	0.008
Diabetes mellitus	266.2	−872.7 to 1405.1	0.647	−208.3	−1700.2 to 1283.6	0.784	115.6	−759.8 to 991.0	0.796
Cardiovascular disease	308.3	−733.8 to 1350.4	0.562	786.9	−726.4 to 2300.1	0.308	412.9	−423.4 to 1249.3	0.333
**Overall social costs**									
Age (>74 years)	76.0	−1346.5 to 1498.5	0.917	−891.3	−2536.3 to 753.6	0.288	−490.0	−1600.9 to 620.9	0.387
Female gender	94.5	−1303.4 to 1492.4	0.895	1634.9	−273.6 to 3543.4	0.093	812.2	−366.4 to 1990.7	0.177
Diabetes mellitus	2229.2	638.8 to 3819.6	0.006	1034.4	−763.1 to 2831.9	0.259	1809.4	620.6 to 2998.2	0.003
Cardiovascular disease	1779.5	356.9 to 3202.2	0.014	2836.8	1008.0 to 4665.5	0.002	2002.6	865.9 to 3139.3	0.001

*GLM* generalized linear model, *CKD* chronic kidney disease, *CI* confidence interval

## Discussion

The increase in the prevalence of CKD with its risk factors and comorbidities such as diabetes and cardiovascular disease as well as the aging of population makes CKD a major issue for healthcare systems [1]. Since 2012, CKD has been included among the pathologies belonging to the “Essential Levels of Care” (Livelli Essenziali di Assistenza) by the Italian Ministry of Health. The Italian NHS has to provide healthcare assistance with or without a patient’s co-payment to CKD patients using public financial resources [30].

Our study reports data from just one region of Italy, i.e. Tuscany, and this could be considered a limitation of the study. In Italy, the provision of health care services reflects the significant socio-economic heterogeneity of the country, being a fully regionalized healthcare system, but the variation observed within a region was often greater than cross-regional variation [31]. Despite the fact that Tuscany has an excellence regional healthcare system [32], our study included centers belonging to both Local Health Authorities and University Hospital trusts distributed throughout Tuscany, representing heterogeneous situations inside the Region. The Italian National Health System has established several mechanisms for improving evenness in the quality of healthcare across its territory [31]. Moreover, the incidence and prevalence of renal replacement therapies are comparable all over the country and the management of chronic kidney disease in adult patients is widely shared [33]. In this framework, the study results of the Tuscany Region on CKD stages 4 and 5 patient costs can be used to describe the Italian picture.

In Italy, awareness about the burden of CKD is less widespread. Before 2016, the survey of the Italian National Institute of Statistics (ISTAT) about the “Health conditions and use of healthcare services” did not mention CKD among the chronic diseases identified as health issues [34]. An Italian cross-sectional study on the detection and awareness of moderate to advanced CKD by primary care practitioners revealed that only 17 % of the patients in charge underwent the serum creatinine testing and, of these, 16.2 % were affected by CKD (GFR <60 mL/min). A nephrology consultation was required by primary care practitioners only in 4.9 % of patients with CKD stage 3 and 55.7 % of patients with severe CKD [35].

The problem of late referral of patients with CKD has been well documented in the scientific literature with a substantial proportion of patients characterized by the short time between the first nephrology evaluation and initiation of dialysis, leading to missed opportunities to improve outcomes by timely management of their kidney disease [36–38].

The low awareness of CKD leads to late diagnosis in the disease’s natural history until dialysis or kidney transplant with great health, social and economic impacts. The adoption of an efficient and integrated patient management system with opportune screening, early taking charge of patients by specialists physicians, and the use of innovative therapies as well as the use of new technologies for in-home care may delay the progression to end-stage renal disease. The presence of patients who are referred late to a nephrologist and who are not currently in the charge of an outpatient clinic could underestimate the burden of CKD in our study.

The economic evaluation of the 4 and 5 pre-dialyses stages is a relevant topic for improving the awareness that the delay in the progression of the disease leads to saving healthcare and economic resources. By considering the only direct medical component, the annual cost of dialyses per patient has been estimated as €34,072 in Italy [39].

To our knowledge, this is the first cost of illness study in Italy aimed at quantifying all the cost items of stages 4 and 5 pre-dialyses adopting a social perspective. Moreover, complete cost of illness estimates are provided by few studies in the international scientific literature, so our study’s results contribute to the knowledge of the economic burden of the disease.

Since studies investigating the cost of pre-dialyses CKD are heterogeneous in terms of stage of disease analyzed, methods, perspective of analysis, target population and country, comparisons between study results have to be made with caution [40]. However, a different burden of hospitalization costs on the total direct medical costs have been found in our study with respect to other studies. Hospitalization costs accounted for 47 and 71 % of the total direct medical costs, respectively, in the USA [41] and in Germany [14] for CKD stages 2–4. In our study, only the 35 % of the total direct medical cost is due to hospitalization costs with a mean number per patient of 3.5 (±7.3) days stay in hospital, revealing an efficient management of the disease. Outpatient services take in charge patients by reducing hospitalizations due to the disease. Among direct medical costs, drugs have the most relevant impact with a mean cost per patient/year of almost €2310. The burden of drug costs on the total social cost of CKD (29 %) is, however, lower than the burden sustained by patients, caregivers and the productivity system estimated by non-medical and indirect costs (44 %).

Some assumptions were made to estimate direct non-medical costs and indirect costs. Among direct non-medical costs, patient cost for transport was estimated starting from the information collected on the CRF regarding the out-of-pocket expenses for a round trip from home to Hospital for performing a specialist visit. In order to estimate the yearly cost per patient for transport, it was assumed that the same money was paid for each specialist visit and for inpatient care and hospital admission in the year of analysis. The transport costs for performing laboratory and diagnostic exams were not considered, and nor for going to the pharmacy, leading to potential underestimations of the data. Expenses incurred by caregivers have been hypothezed as equal to zero in cases of private transport and equal to those of patients in cases of public transport. Real data of patients and caregiver transport expenses should be collected by prospective studies by recording all data at each follow-up; in our study, they have been collected retrospectively leading to a potential recall bias. Regarding the unpaid domestic help provided by caregivers to patients, this has been evaluated with the wage that would have been paid to a housekeeper in accordance with the health economic evaluation literature [23]. This approach could overestimate the real cost of domestic help. Indirect costs have been estimated using the human capital approach only for working patients and caregivers without economically evaluating the leisure time lost by non workers in order to provide a real cost. Sensitivity analyses was then computed by adopting the replacement cost approach for estimating the loss of productivity of patients like housewives and retirees of working age and of non-working caregivers. By considering the replacement cost approach, indirect costs increased from €552.7 ± €2697.6 (estimated in the main analyses) to €2683.3 ± €3325.2.

## Conclusion

By analyzing all cost items, the results reveal that direct non-medical costs and indirect costs were weighted on the social cost of CKD almost as much as the direct medical cost (i.e. 44 % of the overall social cost). Direct non-medical and indirect costs represent the main component of the social cost of CKD (56 % of the overall social cost) in the sensitivity analyses. Patients, their families and the productivity system sustain the burden of the disease almost as much as the healthcare system. By applying the study results to the Italian epidemiological data of the disease [4, 42], it has been estimated that in Italy the overall annual social cost for 227,959 adult patients with CKD stages 4 and 5 pre-dialyses was €1,809,552,398 representing 0.11 % of the Gross Domestic Product (GDP) [43]. Direct costs accounted for €1,692,267,579 (due to the medical component of €1,001,955,049 and the non-medical component of €690,312,531) and indirect costs accounted for €117,284,819.

The study’s results show that economic evaluations quantifying only direct medical costs give a limited view of the phenomenon[44–46]. Assessments of the economic burden of CKD in stages 4 and 5 pre-dialyses have to be based on considering all cost items in order to estimate the real entity of the social costs.

## Electronic supplementary material

Below is the link to the electronic supplementary material.

Supplementary material 1 (DOC 59 kb)
