# Supplementary material for: The social cost of chronic kidney disease in Italy
Source: Eur J Health Econ. 2016 Oct 3;18(7):847–58. doi: 10.1007/s10198-016-0830-1 (PMC5533856; doi:10.1007/s10198-016-0830-1)
Supplement: Supplementary file 1 — Supplementary material 1 (DOC 59 kb) [file 10198_2016_830_MOESM1_ESM.doc]

**Table: Sensitivity Analysis - unadjusted mean social annual cost data per patient by cost component and by CKD stage 4 and 5 pre-dialyses (Euro 2016**)

|  | **CKD stage 4** | | | **CKD stage 5** | | | **Overall** | | | ***p^1^*** |
| --- | --- | --- | --- | --- | --- | --- | --- | --- | --- | --- |
| **Cost component** | **Mean** | **SD** | **%** | **mean** | **SD** | **%** | **mean** | **SD** | **%** |
| **Direct medical cost** |  |  |  |  |  |  |  |  |  |  |
| Laboratory test | 281.5 | ± 222.6 | 3.0% | 392.6 | ± 233.2 | 3.5% | 328.5 | ± 233.4 | 3.2% |  |
| Specialist visit | 101.6 | ± 69.9 | 1.1% | 126.3 | ± 66.6 | 1.1% | 112.1 | ± 69.5 | 1.1% |  |
| Diagnostic exam | 157.4 | ± 167.4 | 1.7% | 186.0 | ± 176.0 | 1.7% | 169.5 | ± 171.5 | 1.7% |  |
| Hospitalization | 1,362.4 | ± 3,250.6 | 14.3% | 1,895.6 | ± 3,100.4 | 17.0% | 1,588.2 | ± 3,195.5 | 15.6% |  |
| Drugs | 2,075.1 | ± 2,016.3 | 21.8% | 2,629.3 | ± 2,173.7 | 23.6% | 2,309.8 | ± 2,100.2 | 22.6 |  |
| Total | 3,978.1 | ± 4,187.0 | 41.8% | 5,229.8 | | ± 4,161.2 | | --- | | 46.9% | 4,508.2 | ± 4,217.5 | 44.2% | *0.001* |
| **Direct non medical cost** |  |  |  |  |  |  |  |  |  |  |
| Out of pocket cost of diet | 154.7 | ± 421.9 | 1.6% | 170.4 | ± 420.7 | 1.5% | 161.3 | ± 421.1 | 1.6% |  |
| Reimbursement of diet | 447.8 | ± 285.4 | 4.7% | 539.0 | ± 207.1 | 4.8% | 486.4 | ± 258.9 | 4.8% |  |
| Transport of patient and caregiver | 60.4 | ± 80.4 | 0.6% | 75.5 | ± 84.5 | 0.7% | 66.8 | ± 82.4 | 0.7% |  |
| Paid domestic help | 701.7 | ± 2,378.0 | 7.4% | 508.3 | ± 2,208.6 | 4.6% | 619.8 | ± 2,307.4 | 6.1% |  |
| Informal care | 1,705.6 | ± 3,516.0 | 17.9% | 1,651.1 | ± 3,458.1 | 14.8% | 1,682.5 | | ±3,488.1 | | --- | | 16.5% |  |
| Total | 3,070.2 | ± 3,984.9 | 32.3% | 2,944.3 | ± 3,958.0 | 26.4% | 3,016.9 | ± 3,969.9 | 29.6% | *0.731* |
| **Indirect costs** |  |  |  |  |  |  |  |  |  |  |
| Loss of productivity of patient | 561.5 | ± 2,412.8 | 5.9% | 1,063.0 | ± 3,541.9 | 9.5% | 773.9 | ± 2,951.4 | 7.6% |  |
| Loss of productivity of caregiver | 1,905.0 | ± 1,666.2 | 20.0% | 1,915.3 | ± 1,622.7 | 17.2% | 1,909.3 | | ±1,646.2 | | --- | | 18.7% |  |
| Total | 2,466.5 | ± 2,765.2 | 25.9% | 2,978.3 | ± 3,950.5 | 26.7% | 2,683.3 | ± 3,325.2 | 26.3% | *0.113* |
|  |  |  |  |  |  |  |  |  |  |  |
| **Overall** | 9,514.8 | ± 6,545.9 | 100.0% | 11,152.4 | ± 7,644.0 | 100.0% | 10,208.4 | ± 7,070.9 | 100.0% | *0.012* |

CKD chronic kidney disease, SD standard deviation.

^1^ Two-sample t-test
